# Piracetam as a Therapeutic Agent for Doxorubicin-Induced Cognitive Deficits by Enhancing Cholinergic Functions and Reducing Neuronal Inflammation, Apoptosis, and Oxidative Stress in Rats

**DOI:** 10.3390/ph15121563

**Published:** 2022-12-14

**Authors:** Vasudevan Mani, Syed Imam Rabbani, Ali Shariq, Palanisamy Amirthalingam, Minhajul Arfeen

**Affiliations:** 1 Department of Pharmacology and Toxicology, College of Pharmacy, Qassim University, Buraydah 51452, Saudi Arabia; 2 Department of Pathology, College of Medicine, Qassim University, Buraydah 51452, Saudi Arabia; 3 Department of Pharmacy Practice, Faculty of Pharmacy, University of Tabuk, Tabuk 47512, Saudi Arabia; 4 Department of Medicinal Chemistry and Pharmacognosy, College of Pharmacy, Qassim University, Buraydah 51452, Saudi Arabia

**Keywords:** piracetam, chemobrain, doxorubicin, acetylcholinesterase, neuroinflammation, apoptosis, oxidative stress

## Abstract

Cancer chemotherapy is known to cause cognitive defects in patients. Our study investigated the effect of piracetam (PIRA; 200 or 400 mg/kg) against doxorubicin (DOX)-induced cognitive deficits in a rat model. The cognitive parameters were analyzed using elevated plus-maze, novel object recognition, and Y-maze tests. Acetylcholinesterase (AChE), neuroinflammatory mediators (cyclooxygenase-2 (COX-2), prostaglandin E2 (PGE2), nuclear factor-κB (NF-κB), tumor necrosis factor-alpha (TNF-α)), apoptotic proteins (B-cell lymphoma-2 (Bcl-2), Bcl2 associated X protein (Bax), cysteine aspartate specific protease-3 (caspase-3)), oxidative parameters (malondialdehyde (MDA), catalase (CAT), and glutathione (GSH)) were also determined in the brain. PIRA administration offered significant protection against DOX-induced cognitive deficits in all maze tests and restored cholinergic functions via a significant reduction in AChE levels. Additionally, PIRA suppressed DOX-induced neuroinflammatory mediators (COX-2, PGE2, NF-κB, and TNF-α), pro-apoptotic proteins (Bax and caspase-3), and oxidative stress (MDA). Besides, it facilitated antioxidant (CAT and GSH) levels. Hence, our study highlighted that the neuroprotective activity of PIRA against DOX-induced cognitive deficits can be linked to reductions of AChE levels, neuro-inflammatory mediators, pro-apoptotic proteins, and oxidative stress.

## 1. Introduction

Piracetam (PIRA) is chemically called 2-Oxo-1-pyrrolidine-acetamide and is a cyclic derivative of γ-aminobutyric acid (GABA). The drug was first approved in 1971 for treating vertigo and other conditions related to age factors [1]. Several studies conducted in the past have indicated that the administration of PIRA improved electro-convulsive, pentylenetetrazole-induced seizures, age-induced dementia, and cognitive impairment in animal models [2]. Recently, Verma et al. (2018) explained the beneficial mechanisms of PIRA in neurodegenerative disease. Treatment with PIRA has shown a neuroprotective effect against lipopolysaccharide (LPS)-induced neuroinflammation by attenuating inflammatory cytokines and astrocytes activation in rats [3]. It showed significant protection against LPS-induced ROS and depleted mitochondrial membrane potential. The anti-apoptotic activity of PIRA was also established against LPS-induced cellular death in caspase-independent pathways [3,4]. Although the precise mechanism for the nootropic activity is not reported in the literature, the influence of the treatment on the conversion of ADP to ATP, improvement in neuronal phospholipase A2 activity, and enhanced acetylcholine (ACh) utilization in the brain have been linked to cognitive improvement activity [1,5]. Re-organization of disturbed membrane lipids, monoamines, and neurotransmitter levels has also been reported for the mechanism of nootropic activity of PIRA [3].

Doxorubicin (DOX) is a derivative of anthracycline and was first extracted from *Streptomyces pencetius* vas. Caesius. The drug is reported to be used therapeutically in the treatment of various cancers listed as breast, gastric, lung, ovarian, thyroid, Hodgkin’s lymphoma, non-Hodgkin’s, sarcoma, multiple myeloma, and pediatric cancers [6]. The anti-cancer activity of the drug is mediated through the following mechanism; intercalation of the DNA, interruption of topoisomerase-II arbitrated DNA repair, and production of free radicals resulting in damage to cellular components, including nucleic acid [7]. Administration of DOX is known to cause several adverse effects ranging from mild to severe intensity [8]. The significant long-term complications of DOX chemotherapy are reported to be cardiotoxicity and chemobrain. Given the higher incidences of cancer reported worldwide, these defects need to be suitably redressed to enhance the compliance of cancer chemotherapy [9].

According to the literature, ‘chemobrain’ can be defined as cognitive deficits that could occur due to defects in processing speed, memory retention, and concentration during the course of chemotherapy treatment [10]. Although the precise mechanism of this type of neurological defect is not clearly understood, inflammatory responses associated with chemotherapeutic agents are strongly implicated. This defect in brain function is a matter of concern for both patients and healthcare providers [11]. An estimated 13–78% of cancer patients were reported to suffer from this drug-induced complication. This neurological complication was found to interfere with both prognoses as well as the therapeutic outcome in patients treated with cancer chemotherapy [12].

Previous studies have indicated that the cytotoxic activity of DOX in cancer chemotherapy can activate several inflammatory and pro-inflammatory processes. These activities have been linked to a disturbed regulation of cytokines functions [13]. Furthermore, the triggering of the apoptosis pathway is believed to be an important mechanism associated with DOX-related cognitive defects. In primary cortical neurons, it was reported that DOX-induced cellular apoptosis was dependent on an extrinsic mechanism or “death receptor-mediated” apoptosis [14]. Additionally, DOX containing a quinone molecule was reported to induce oxidative stress in tissues by elevation of hydroxyl (•OH) as well as hydrogen peroxide (H_2_O_2_) radicals, and via O2•-production. The oxidative stress results in excessive ROS production, leading to the modification of the molecular protein, lipid, and nucleic acids [15]. 

Several attempts have been made in the past to reduce these complications of DOX. Recently, berberine, Kai-Xin-San, astaxanthin, morin, and polydatin were found to improve the parameters that are indicative of memory enhancement in DOX-treated animal models [16,17,18,19,20]. These targeted lead compounds are linked to several protective mechanisms against DOX-induced neuronal inflammation, oxidative stress, apoptosis, and neural degeneration. However, the efficacy of drugs on chemobrain is still under various stages of testing. Our recent data highlighted that treatment of levetiracetam protected the DOX-induced cognitive deficits and neuroinflammation in rats [21]. PIRA, being the first approved nootropic agent, has also been reported to possess cholinergic activation, anti-inflammatory, antioxidant, and anti-apoptosis properties [2,3]. Hence, the present study was planned to evaluate the efficacy of PIRA on DOX-induced cognitive impairment and determine its role in the mediators of cholinergic, neuroinflammation, apoptosis, and oxidative insults in the DOX-induced experimental rat model.

## 2. Results

### 2.1. Effect of PIRA on DOX-Induced Cognitive Impairment Parameters Using an Elevated Plus-Maze (EPM) Test

**Figure 1:**
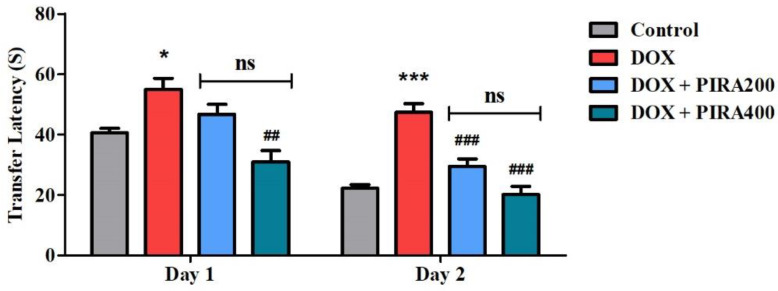
Effect of piracetam (PIRA) on day 1 and day 2 transfer latency (s) of doxorubicin (DOX)-induced rats using an elevated plus-maze test. The results are expressed by mean ± SEM (*n* = 7). A one-way ANOVA [*F*(3,24) = 10.20, *p* < 0.001 for day 1 and *F*(3,24) = 26.96, *p* < 0.001 for day 2 of the EPM test] was conducted, followed by a Tukey–Kramer multiple comparisons test. * *p* < 0.05, and *** *p* < 0.001 as compared to the control group; ns—not significant as compared to the control group; ^##^ *p* < 0.01 and ^###^ *p* < 0.001 as compared to the DOX-induced group.

The effect of PIRA (200 and 400 mg/kg, p.o.) on DOX-induced spatial memory impairment using EPM is represented in Figure 1. The statistical analysis of the results on day 1 indicated that DOX treatment significantly increased (*p* < 0.05; 55.00 ± 3.62S) the transfer latency (TL) when compared with the control group (40.57 ± 1.49S). PIRA at the highest tested dose (400 mg/kg) was found to reduce the TL (*p* < 0.01; 30.86 ± 3.78S) in comparison with the DOX group. Further, the analysis of the day 2 results revealed that DOX treatment enhanced the TL (*p* < 0.001; 47.43 ± 2.85S) extensively compared to the control group (22.14 ± 1.24S). Besides, both the tested doses of PIRA (200 and 400 mg/kg) significantly diminished the TL period (*p* < 0.001; 29.43 ± 2.50S and 20.14 ± 2.64S, respectively) in the EPM test when a comparison was done with the DOX group.

### 2.2. Effect of PIRA on DOX-Induced Cognitive Impairment Parameters Using Novel Object Recognition (NOR) Test

**Figure 2:**
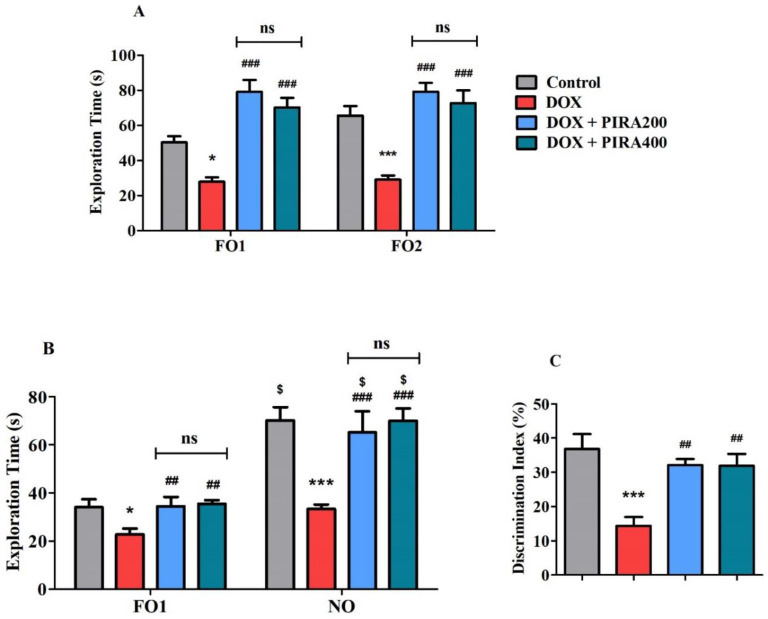
Effect of piracetam (PIRA) on (**A**) exploration time of two familiar objects (FO1 and FO2) during the training session, (**B**) exploration time of familiar (FO1) and novel (NO) objects during the test session, and (**C**) discrimination index of doxorubicin (DOX)-induced rats model using a novel object recognition test. The results are expressed by mean ± SEM (*n* = 7). A one-way ANOVA [*F*(3,24) = 21.92, *p* < 0.001 for FO1 and *F*(3,24) = 17.90, *p* < 0.001 for FO2 during a training session; *F*(3,24) = 8.805, *p* < 0.001 for FO1 and *F*(3,24) = 20.42, *p* < 0.001 for NO during test session; *F*(3,24) = 9.829, *p* < 0.001 for DI] was conducted, followed by a Tukey–Kramer multiple comparisons test for comparisons of within the groups. The student’s unpaired *t*-test was used to compare each group’s exploration time. ^$^ *p* < 0.001 as compared to the corresponding group; * *p* < 0.05, and *** *p* < 0.001 as compared to the control group; ns—not significant as compared to the control group; and ^##^ *p* < 0.01, and ^###^ *p* < 0.001 as compared to the DOX-induced group.

The NOR test was performed to evaluate the effect of PIRA on various cognitive behaviors focused on recognition memory in DOX-induced rats (Figure 2). During the training session (Figure 2A), when both similar objects (familiar objects; FO1 and FO2) were used, considerable decreases were noted in the exploration time of both FO1 (*p* < 0.05; 27.86 ± 2.51S) and FO2 (*p* < 0.001; 29.14 ± 2.33S) with DOX-induced animals when compared to the corresponding control groups (50.29 ± 3.49S for FO1 and 65.57 ± 5.43S for FO2). However, the administration of PIRA (200 and 400 mg/kg, p.o.) led to a significant improvement in exploration time (*p* < 0.001) of both FO1 (79.14 ± 6.70S and 70.14 ± 5.60S, correspondingly) and FO2 (79.29 ± 4.94S and 72.71 ± 7.27S, correspondingly) in DOX-induced rats in relation to the specific DOX group. Moreover, there were no considerable differences between the exploration time of both objects as well as compared with respective control groups.

During the test session (Figure 2B), when a familiar object (FO2) was exchanged with a targeted novel object (NO), the DOX induction reduced the exploration of the corresponding control groups significantly (34.00 ± 3.38S for FO1 and 70.14 ± 5.54S for NO). However, the exploration time of FO1 improved considerably (*p* < 0.01; 39.86 ± 1.67S for 200 mg/kg and 35.43 ± 1.53S for 400 mg/kg) with the treatment of PIRA when compared to the DOX-induced group. Besides, the comparison of the exploration time of NO highlighted that both doses of PIRA (200 and 400 mg/kg, p.o.) enhanced the exploration time (*p* < 0.001; 77.86 ± 3.64S and 70.00 ± 5.15S, respectively) as compared to the respective DOX-induced animals. Interestingly, the comparison of the exploration time between the two objects FO1 and NO, excluding the DOX-induced group, control as well as PIRA (200 and 400 mg/kg, p.o.) treatment groups resulted in an improvement of exploration time (*p* < 0.001) in comparison with respective groups of FO1.

In continuation, the percentage of discrimination index of each group was calculated (Figure 2C) to explain the effect of DOX and PIRA treatment on the discrimination ability of animals between the two objects FO1 and NO during the test session. A significant reduction in discrimination index value (*p* < 0.001; 14.36 ± 2.53%) was recorded with DOX-induced rats in comparison with control rats (36.84 ± 4.36%). Yet, the groups of animals treated with both doses of PIRA (200 and 400 mg/kg, p.o.) successfully improved the discrimination index values (*p* < 0.01; 32.15 ± 1.75% and 31.96 ± 3.39%, correspondingly) in comparison with the DOX-induced animals.

### 2.3. Effect of PIRA on DOX-Induced Cognitive Impairment Parameters Using Y-Maze Test

**Figure 3:**
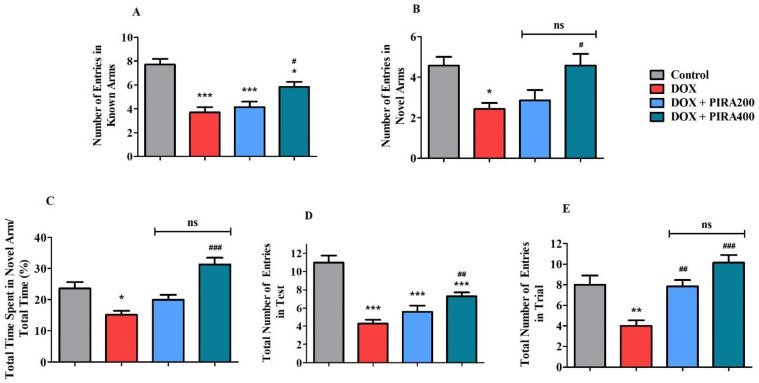
Effect of piracetam (PIRA) on (**A**) the number of entries in known arms in the test session, (**B**) the number of entries in the novel arm in the test session, (**C**) the percentage of time spent in the novel arm in the test session, (**D**) the total number of entries in the test, and (**E**) the total number of entries in the trial session of the doxorubicin (DOX)-induced rat model using a Y-maze. The results are expressed by mean ± SEM (*n* = 7). A one-way ANOVA [*F*(3,24) = 17.60, *p* < 0.001 for the number of entries in the known arms; *F*(3,24) = 5.929, *p* < 0.01 for the number of entries in a novel arm; *F*(3,24) = 14.43, *p* < 0.001 for the percentage of time spend in novel arm; *F*(3,24) = 24.37, *p* < 0.001 for the total number of entries in the test; *F*(3,24) = 13.12, *p* < 0.001 for the total number of entries in the trial] was conducted, followed by a Tukey–Kramer multiple comparisons test. * *p* < 0.05, ** *p* < 0.01, and *** *p* < 0.001 as compared to the control group; ns—not significant as compared to the control group; ^#^ *p* < 0.05, ^##^ *p* < 0.01, and ^###^ *p* < 0.001 as compared to the DOX-induced group.

Figure 3 represents the results of the PIRA effect on DOX-treated animals in a Y-maze. In the test session, analysis of the data indicated that DOX treatment reduced the number of entries (*p* < 0.001; 3.71 ± 0.42) in the known arms of the Y-maze as compared to the control group (7.71 ± 0.47). Administration of PIRA at a lower tested dose (200 mg/kg) did not significantly alter the number of entries but a higher dose of the PIRA (400 mg/kg) increased it (*p* < 0.05; 5.86 ± 0.40) when compared with the DOX group (Figure 3A). Similarly, the number of entries in the novel arm was found to be reduced (*p* < 0.05; 2.43 ± 0.30) in DOX-treated animals as compared to the control animals (4.57 ± 0.43). In the treatment group, only the higher dose of PIRA (400 mg/kg) was observed to increase the number of entries (*p* < 0.05; 4.57 ± 0.57) when compared with DOX-treated animals (Figure 3B). The total percentage of time spent in the novel arm was found to be reduced (*p* < 0.05; 15.09 ± 1.28%) in DOX-treated animals in comparison with the normal group (23.57 ± 2.06%). PIRA at 400 mg/kg enhanced the total time spent percentage (*p* < 0.001; 31.33 ± 2.13%) when compared with the DOX-treated animals. A lower dose of PIRA (200 mg/kg) did not produce any significant variation (Figure 3C).

The total number of entries in the test session was found to be reduced (*p* < 0.001; 4.29 ± 0.42) in DOX-treated animals in comparison with the control group (11.00 ± 0.76). Oral PIRA treatment at 400 mg/kg was found to enhance these entries considerably (*p* < 0.01; 7.29 ± 0.42) compared to the DOX group. Further, lower (200 mg/kg) and higher (400 mg/kg) doses of PIRA also showed a reduction (*p* < 0.001) in the total number of entries as compared with the control animals (Figure 3D). In the trial session, the total number of entries was found to be reduced (*p* < 0.01; 4.00 ± 0.53) in DOX-treated animals when compared to the control rats (8.00 ± 0.90). An improvement in the total number of entries was recorded for both the lower (*p* < 0.01; 7.86 ± 0.59) and higher (*p* < 0.001; 10.14 ± 0.74) doses of PIRA when compared with DOX-treated animals’ data (Figure 3E).

### 2.4. Effect of PIRA on Acetylcholinesterase Level in the Brain Homogenate of DOX-Treated Animals

**Figure 4:**
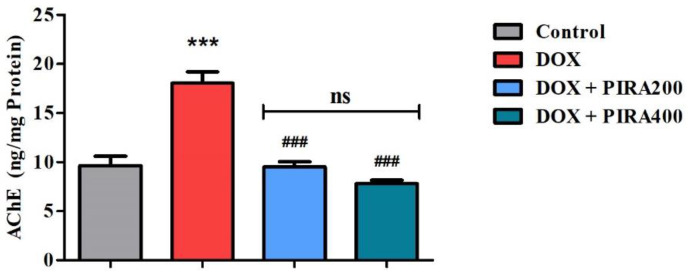
Effect of piracetam (PIRA) on acetylcholinesterase (AChE) levels in brain homogenates of the doxorubicin (DOX)-induced rat model. The results are expressed by mean ± SEM (*n* = 7). A one-way ANOVA [*F*(3,24) = 31.87, *p* < 0.001] was conducted, followed by a Tukey–Kramer multiple comparisons test. *** *p* < 0.001 as compared to the control group; ns—not significant as compared to the control group; ^###^ *p* < 0.001 as compared to the DOX-induced group.

The data from the estimation of acetylcholinesterase (AChE) from the brain homogenate of the animals indicated that DOX treatment increased the enzyme levels (*p* < 0.001; 9.66 ± 0.95 ng/mg protein) when compared with the control group (18.04 ± 1.17 ng/mg protein). Oral administration of PIRA at both the tested doses (200 and 400 mg/kg) was found to decrease the AChE levels (*p* < 0.001; 9.54 ± 0.49 ng/mg protein and 7.80 ± 0.39 ng/mg protein) in brain homogenate when compared to the DOX-treated values (Figure 4).

### 2.5. Effect of PIRA on Neuro-Inflammatory Mediators in the Brain Homogenate of DOX-Treated Animals

**Figure 5:**
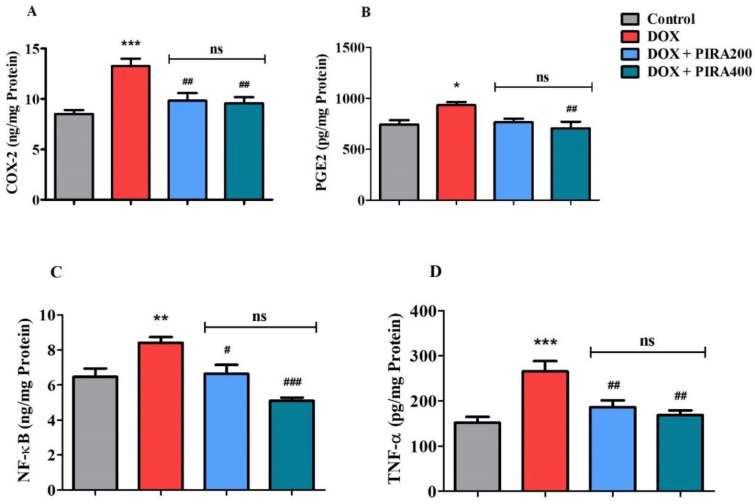
Effect of piracetam (PIRA) on inflammatory parameters (**A**) COX-2, (**B**) PGE2, (**C**) NF-κB, and (**D**) TNF-α, in doxorubicin (DOX)-induced rat model. The results are expressed by mean ± SEM (*n* = 7). A one-way ANOVA [*F*(3,24) = 10.65, *p* < 0.001 for COX-2; *F*(3,24) = 5.105, *p* < 0.01 for PGE2; *F*(3,24) = 12.26, *p* < 0.001 for NF-κB; *F*(3,24) = 9.858, and *p* < 0.001 for TNF-α] was conducted, followed by a Tukey–Kramer multiple comparisons test. * *p* < 0.05, ** *p* < 0.01, and *** *p* < 0.001 as compared to the control group; ns—not significant as compared to the control group; ^#^ *p* < 0.05, ^##^ *p* < 0.01 and ^###^ *p* < 0.001 as compared to the DOX-induced group.

Analysis of neuro-inflammatory biomarkers levels such as cyclooxygenase-2 (COX-2), prostaglandin E2 (PGE2), nuclear factor-κB (NF-κB), and tumor necrosis factor-alpha (TNF-α) is summarized in Figure 5. DOX treatment in the animals was observed to elevate the levels of COX-2 enzymes (*p* < 0.001; 13.26 ± 0.73 ng/mg protein) when compared to the control group (8.54 ± 0.36 ng/mg protein). The tested doses of PIRA (200 and 400 mg/kg, p.o.) showed a considerable reduction (*p* < 0.01; 9.85 ± 0.74 ng/mg protein for 200 mg/kg and 9.56 ± 0.61 ng/mg protein for 400 mg/kg) in the brain tissues’ COX-2 levels when the data was compared with the DOX-treated animals (Figure 5A). The estimation of PGE2 levels in the brain homogenate was found to be enhanced (*p* < 0.05; 933.8 ± 30.33 pg/mg protein) in DOX-treated animals in comparison with the control group (744.1 ± 41.90 pg/mg protein). Treatment of PIRA at a higher dose (400 mg/kg) diminished the enzyme levels (*p* < 0.01; 706.8 ± 63.61 pg/mg protein) when compared with the DOX values (Figure 5B).

Further, the NF-κB levels were found to be elevated (*p* < 0.01; 8.42 ± 0.32 ng/mg protein) in DOX-treated animals as compared to the control group (6.48 ± 0.46 ng/mg protein). Administration of PIRA at 200 mg/kg decreased the level of the enzymes (*p* < 0.05; 6.65 ± 0.50 ng/mg protein) in comparison with the DOX-treatment group. The effect of PIRA on brain NF-κB levels was found to be further reduced (*p* < 0.001; 5.11 ± 0.18 ng/mg protein) when the drug was tested at a higher dose (400 mg/kg) (Figure 5C). Similarly, the estimation of brain TNF-α indicated an increase in enzyme levels (*p* < 0.001; 265.6 ± 22.59 pg/mg protein) when compared to the control group (151.7 ± 12.84 pg/mg protein). PIRA at both the tested doses (200 and 400 mg/kg) was observed to reduce the TNF-α levels in the brain homogenate (*p* < 0.01; 185.9 ± 15.79 pg/mg protein and 168.8 ± 10.28 pg/mg protein, respectively) when compared with the DOX-treated group (Figure 5D).

### 2.6. Effect of PIRA on Apoptosis Parameters in the Brain Homogenate of DOX-Treated Animals

**Figure 6:**
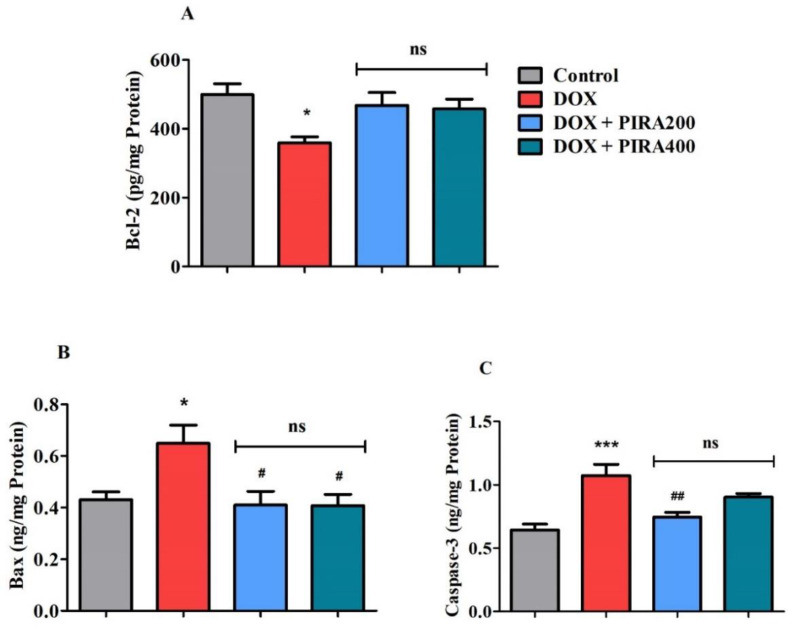
Effect of piracetam (PIRA) on apoptosis parameters (**A**) Bcl-2, (**B**) Bax, and (**C**) caspase-3 in doxorubicin (DOX)-induced rat model. The results are expressed by mean ± SEM (*n* = 7). A one-way ANOVA [*F*(3,24) = 5.099, *p* < 0.01 for Bcl-2; *F*(3,24) = 4.160, *p* < 0.05 for Bax; *F*(3,24) = 11.43, *p* < 0.001 for caspase-3] was conducted, followed by a Tukey–Kramer multiple comparisons test. * *p* < 0.05, and *** *p* < 0.001 as compared to the control group; ns—not significant as compared to the control group; ^#^ *p* < 0.05 and ^##^ *p* < 0.01 as compared to the DOX-induced group.

Figure 6 shows the results of targeted apoptosis parameters B-cell lymphoma-2 (Bcl-2), Bcl2 associated X protein (Bax), and caspase-3 levels represented in brain homogenate of DOX- and PIRA-treated rats. The DOX treatment caused a decline in the level of an anti-apoptosis marker Bcl-2 (*p* < 0.05; 359.2 ± 17.41 pg/mg protein) as compared with the control animals (499.3 ± 32.15 pg/mg protein) (Figure 6A). The PIRA treatment did not lead to any significant changes in Bcl-2 levels (468.5 ± 37.02 pg/mg protein for 200 mg/kg and 457.6 ± 28.70 pg/mg protein for 400 mg/kg) in DOX-induced rats’ brains.

On the other hand, pro-apoptosis marker Bax levels (Figure 6B) in the brain were elevated (*p* < 0.05; 0.649 ± 0.070 ng/mg protein) in DOX-induced animals as compared to the control rats (0.430 ± 0.030 ng/mg protein). However, the concurrent thirty days administration of PIRA (200 and 400 mg/kg, p.o.) considerably reduced the Bax levels in the brain (*p* < 0.05; 0.410 ± 0.053 ng/mg protein and 0.408 ± 0.044 ng/mg protein) when compared to the DOX-induced group.

Similarly, the levels of caspase-3 (Figure 6C), considered a pro-apoptosis marker, were also elevated (*p* < 0.001; 1.073 ± 0.089 ng/mg protein) in DOX-induced animals’ brains when compared to the control animals (0.643 ± 0.048 ng/mg protein). It was found that administration of PIRA at 200 mg/kg resulted in a reduction of brain caspase-3 levels (*p* < 0.01; 0.746 ± 0.037 ng/mg protein) as compared to the DOX-induced brain. The group of rats treated with a high dose of PIRA (400 mg/kg, p.o.) did not display any significant alteration in brain caspase-3 levels (0.903 ± 0.029 ng/mg protein) in the DOX-induced brain.

### 2.7. Effect of PIRA on Oxidative Parameters in the Brain Homogenate of DOX-Treated Animals

**Figure 7:**
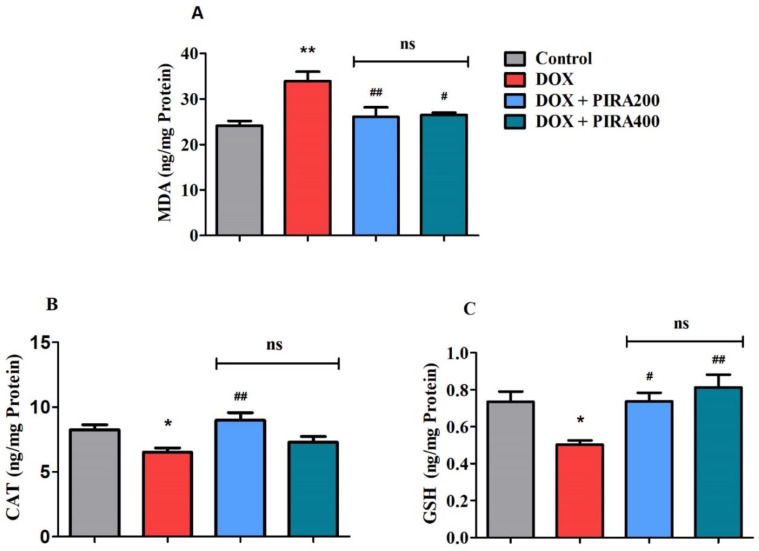
Effect of piracetam (PIRA) on oxidative parameters (**A**) MDA, (**B**) CAT, and (**C**) GSH in doxorubicin (DOX)-induced rat model. The results are expressed by mean ± SEM (*n* = 7). A one-way ANOVA [*F*(3,24) = 7.460, *p* < 0.01 for MDA; *F*(3,24) = 5.939, *p* < 0.01 for CAT; *F*(3,24) = 6.789, *p* < 0.01 for GSH] was carried out, followed by a Tukey–Kramer multiple comparisons test. * *p* < 0.05, and ** *p* < 0.01 as compared to the control group; ns—not significant as compared to the control group; ^#^ *p* < 0.05 and ^##^ *p* < 0.01 as compared to the DOX-induced group.

The effect of four repeated doses of DOX-induction and thirty days of PIRA treatment on the levels of various oxidative parameters in brain tissues malondialdehyde (MDA), catalase (CAT), and glutathione (GSH) levels is shown in Figure 7. The selective oxidative stress marker MDA (Figure 7A) was found to be elevated (*p* < 0.01; 33.16 ± 2.05 ng/mg protein) in DOX-induced brains as compared to control rats (24.15 ± 1.02 ng/mg protein). However, the groups of rats treated with PIRA reversed the MDA levels (*p* < 0.01; 26.07 ± 2.12 ng/mg protein for 200 mg/kg and 26.52 ± 0.49 ng/mg protein for 400 mg/kg) in the brain as compared to DOX-induced rats.

In contrast, the levels of both CAT (Figure 7B) and GSH (Figure 7C), which were targeted antioxidant biomarkers, were decreased (*p* < 0.05; 6.51 ± 0.33 ng/mg protein for CAT and 0.503 ± 0.023 ng/mg protein for GSH) in DOX-induced rat brains as compared to the respective control animals (8.26 ± 0.39 ng/mg protein for CAT and 0.734 ± 0.056 ng/mg protein for GSH). Interestingly, it was found that treatment with a lower dose of PIRA (200 mg/kg, p.o.) elicited a higher level of CAT (*p* < 0.01; 8.99 ± 0.58 ng/mg protein) in the brain as compared to the DOX-induced group. Additionally, another antioxidant marker in the brain, GSH, was also elevated by the treatment of PIRA at 200 mg/kg (*p* < 0.05; 0.737 ± 0.047 ng/mg protein) and 400 mg/kg (*p* < 0.01; 0.813 ± 0.069 ng/mg protein) when compared to DOX-induced rats.

## 3. Discussion

DOX is one of the most frequently used drugs in the chemotherapy of several types of cancers [7]. DOX treatment in cancer patients has been reported to induce both short-term as well as long-term memory impairment [22]. Although cardiotoxicity is considered to be the main complication of DOX chemotherapy, damage to the integrity of patients’ blood–brain barrier is reported to facilitate the entry of the drug into the untargeted sites of the brain. Inflammatory responses and hyperactivity of several types of cytokines are considered the primary cause of altering the functional integrity of the blood–brain barrier [23]. In addition, long-term administration of DOX is reported to affect the microbiota in cancer patients, which in turn can dysregulate the cytokines functions [24,25]. All these changes are related to the neurodegeneration of brain cells leading to cognitive impairment. Consistent with this, the present study highlighted that continuous administration of PIRA ameliorates the DOX-induced cognitive impairments, recovers the cholinergic neuronal functions, and protects from neuronal inflammation, apoptosis, and oxidative insults in an experimental rat model.

PIRA, a cyclic derivative of GABA was tested in two doses (200 and 400 mg/kg) against DOX-induced cognitive impairment. It is a well-established cognitive enhancer, which is used as a reference standard for many experimental models. EPM is considered a neutral behavioral model and was employed in this study to assess the rat’s behavior and cognitive abilities [26,27,28]. The data from the results indicated that the higher dose (400 mg/kg) was more effective in the improvement of TL than the lower dose (200 mg/kg) in DOX-induced animals. In addition, retention of the learned task memory assessment (TL) on the second day emphasized the reversal of DOX-induced cognitive deficits through the administration of PIRA 200 and 400 mg/kg. The capabilities of PIRA around DOX-induced deficits in working memory and discrimination ability of rats were extensively analyzed using the NOR test. During the training session, each of the animals was allowed to explore two identical objects (FO1 and FO2) considered familiar objects to allow them to remember, as a part of assessing their working memory [28]. The improvements in the exploration time by animals with the administration of PIRA as compared to DOX induction highlighted the capacity of treated animals to remember the similarity of objects in their working memory. Still, there was no notable difference between FO1 and FO2’s exploration times, meaning that neither PIRA nor DOX induction changed the animal’s ability to recall the similarity of the same objects in our investigation. Furthermore, in the continuation of the NOR test session, while exploring the two different objects (FO1 and NO), the treatment of PIRA (200 and 400 mg/kg, p.o.) led to an increase in the exploration time of NO as compared to the respective exploration time of FO1. It appears that animals preferred to spend more time with a novel target than with a familiar one, indicating that they were able to retain and distinguish both objects as well as remember the FOs from the earlier training. Additionally, the same treatment also exhibited a higher exploration time of FO1 and NO as related to the corresponding DOX-induced group, evidencing the protection of DOX-induced cognitive deficits by PIRA. Moreover, the percentage of discrimination index was calculated for each group to provide further evidence of the animals’ discrimination ability between two targeted objects like FO1 and NO. Our results proved that groups of animals treated with PIRA had higher discrimination index values than DOX-induced animals. A previous report evidenced that superior cognitive abilities are required for the NOR test in order to discriminate unfamiliar things from familiar ones or to complete a task in a novel environment [29]. Our present results are consistent with our prior studies, suggesting that when animals are allowed to move between NO and FO, they commonly approach and prefer to spend more time exploring the unfamiliar NO [26,28].

Another model, the Y-maze test was deployed to assess the effect of PIRA on the spatial working memory of DOX induction. As Liet et al. (2015) have shown, the Y-maze performance by animals is connected with most of their brain areas, like the hippocampus, basal forebrain, and prefrontal cortex [30]. In the present experiment, the Y-maze test was performed in two sessions, trial and test, with an interval of four hours between sessions [26,28]. Initially, in the trial session, the animals were allowed to explore only two arms while one arm (the novel arm) was kept closed. In the test session, all the arms were kept open, so the animals could freely explore all the arms. In general, the prefrontal cortex functions are highlighted by the animals’ tendency to enter into the novel arm frequently in contrast to the familiar arms, which were visited previously in the trial session [31]. According to our results, DOX-induced spatial memory deficits were indicated by a considerable reduction in the number of both known and novel arms entries. In contrast, PIRA treatment at a high dose (400 mg/kg, p.o.) achieved an improvement in the number of known as well as novel arm entries as referenced against the corresponding DOX-induced group, highlighting the improvement in spatial memory. Additionally, enhancement of the percentage of total time spent in the novel arm at a high dose of PIRA (400 mg/kg, p.o.) in DOX-induced rats explained the improvement of animals’ coping behavior in several environments. Interestingly, the improvement in coping behavior is furthermore related to the antianxiety behavior of animals [32]. In addition, PIRA treatment (400 mg/kg, p.o.) led to an improvement in the total number of entries in trial and test sessions as compared to DOX-induce rats. This is directly connected to the enhancement of the curiosity behavior of the animals [33].

Adding support to the above maze results related to cognitive efficacy, the thirty-day treatment with PIRA facilitated cholinergic transmission in the brain through reduced elevated AChE levels in the DOX-induced group. On the other hand, the neurodegenerative effect of DOX can be observed in the present study as the DOX-induced animals were found to have elevated levels of AChE compared with the control group. AChE is an enzyme involved in the hydrolysis of ACh. It is well-evidenced that the brain’s ACh levels, particularly in the hippocampus area, have a vital role in maintaining cognitive functions; lowering the levels of ACh in the hippocampus results in age-related cognitive function impairments [34]. In earlier studies, increased AChE levels have been linked to a decline in brain ACh levels as well as impaired memory functions. The level of enzymes was also used to analyze the extent of neurodegeneration induced by a disease state [26,35]. Moreover, AChE influences the neuroinflammatory response, neuronal apoptosis, oxidative vulnerability, and the aggregation of pathogenic proteins, contributing significantly to the pathogenesis of neurodegenerative disorders. An increase in AChE levels results in decreased levels of ACh and interleukin (IL)-10 at the same it is associated with increased levels of TNF-α, IL-18, IL-12, IL-17, IL-1β, and INF-γ [36]. Our results displayed that it reduced the TNF-α levels with PIRA treatment.

The activation of several neuroinflammatory mediators in the brain homogenate of the animals treated with DOX is evident in the present study. Estimation of COX-2, PGE2, NF-κB, and TNF-α suggested an elevation in the DOX-treated group compared to the control. These observations follow the earlier studies, where DOX administration activated the neuroinflammatory mediators in the experimental set-up [37]. In earlier studies, PIRA exhibited neuroprotective activity in cocaine-induced neuro-epigenetic modification [38]. The data from the present study also indicated that PIRA treatment significantly reduced the levels of COX-2, PGE2, NF-κB, and TNF-α in the brain homogenate of DOX-treated animals. COX-2 is an inducible form of cyclooxygenase and is known to catalyze the conversion of arachidonic acid to prostaglandins [39]. PGE2 plays a vital role in the inflammatory process since it causes direct vasodilation and also activates several other mediators of inflammation [40]. NF-κB is a family of highly conserved transcriptional factors that regulate several biological functions including inflammation [41]. The transcriptional factor NF-κB is also considered redox-sensitive and could be activated by reactive oxygen species (ROS) production in DOX-induction through an IκB kinase-dependent pathway [42,43]. Then, the activation of NF-κB results in promoting the expression of several cytokines including TNF-α and IL-1 [44]. On the other hand, TNF-α is a pro-inflammatory cytokine and causes vasodilation, edema formation, and adhesion of leucocytes to the epithelium [45]. Administration of DOX results in the stimulation of TNF-α synthesis in peripheral tissues, which elevates its level in blood circulation. Through the receptor-mediated endocytosis mechanism in the blood–brain barrier, TNF-α enters into the brain tissues and initiates the neuroinflammatory process by further production of TNF-α in the brain. Furthermore, TNF-α levels activate brain glial cells and facilitate the local production of other pro-inflammatory mediators like NF-κB, IL-1β, and IL-6 [46]. The findings suggest the high potential of treatment with PIRA at 400 mg/kg for minimizing the neuro-inflammatory mediator levels elevated by DOX.

An increased level of LPO has been reported in DOX-treated mouse brains as well as plasma, and also the results extended to the reduction of non-enzymatic and enzymatic antioxidants in the brain areas [47]. The current study indicated that four doses of DOX caused an elevation in MDA levels and declined CAT as well as GSH levels in brain tissues. MDA is the main and most researched by-product of polyunsaturated fatty acid peroxidation and its level is directly increased with oxidative stress. It is considered a marker of LPO and results in potent mutagenic and atherogenic effects by interacting with DNA and various targeted proteins [48]. According to our results, both doses of PIRA (200 and 400 mg/kg, p.o.) treatment successfully lowered MDA levels in the DOX-induced brain. An essential antioxidant enzyme CAT converts cellular hydrogen peroxide into water and oxygen, significantly lowering oxidative stress. The enzyme is involved in the primary antioxidant defensive systems by deactivating and removing ROS in biological macromolecules that protect them from oxidative damage [49]. Our results showed that the CAT enzyme levels in DOX-induced rats’ brains were improved with PIRA (200 mg/kg, p.o.) treatment. In general, a decline in GSH levels or the GSH/GSSG ratio can indicate an excessive amount of ROS. The level of GSH refers to the conversion of glutathione disulfide (GSSG) to GSH by the glutathione reductase enzyme [50]. In the present study, treatment with PIRA (200 and 400 mg/kg, p.o.) restored the GSH levels in a dose-dependent manner in DOX-induced rats’ brains.

Furthermore, the triggering of the apoptosis pathway is believed as an important mechanism associated with DOX-related cognitive defects. In the apoptosis process, Bax acts as a pro-apoptotic member from the Bcl-2 gene family that opens pores present in the outer membrane of cellular mitochondria and releases cytochrome c which activates the apoptosis process. The released cytochrome c triggers caspase and leads to subsequent cell damage [51]. Caspases play a significant role in the apoptotic response. Once caspase-3 is activated, it triggers proteolytic degradation of the majority of cellular targets and resulting in cell death [15]. Interestingly, a cellular protein Bcl-2 protects the opening of the pore in the membrane and leads to anti-apoptotic effects [52]. Additionally, induction of TNF-α by DOX activates caspase pathways by binding on cellular TNF-related apoptosis-inducing ligand (TRAIL) and can cause cellular death through activation of caspase pathways [53]. Furthermore, the reduction of TNF-α levels as well as oxidative stress in brain tissue by a xanthone derivative from *Garcinia mangostanar* resulted in a decrease in the level of BAX and caspase 3 in DOX-induced mice [54]. In the present observation, both the pro-apoptosis proteins such as Bax and caspase-3 levels in the brain were triggered with DOX-induction, and their effects were successfully suppressed by the treatment with PIRA. In contrast, an anti-apoptosis protein Bcl-2 level was reduced in DOX-induced rats, but there was no significant modification with both doses of PIRA treatments. Early treatment of PIRA restored the elevated caspase-3 levels and morphological alteration of neurons and lowered neuronal density which was induced by lipopolysaccharides in rats [3].

## 4. Materials and Methods

### 4.1. Experimental Animals

The use of the animals and experimental protocols of the present study received ethical approval from the Research Center of the College of Pharmacy (Approval ID 2020—CP—14) and the Deanship of Scientific Research of Qassim University under the grant number 10223-pharmacy-2020-1-3-I. A total of 28 adult Sprague Dawley rats (male; 3 months old; body weight 150–200 g) were divided into four groups, each comprising seven animals. The animals were stored in the animal house facility of the College of Pharmacy as per the standard guidelines. The animals were kept in conventional laboratory conditions (room temperature 22 ± 2 °C, 60–70% humidity, 12 h light-dark cycle) for the first week of the acclimatization and the duration of the study. The rodents were fed normal rodent pellet meal (First Milling Company, Jeddah, Saudi Arabia) and were given free access to water.

### 4.2. Experimental Groups and Drug Treatment

**Figure 8:**
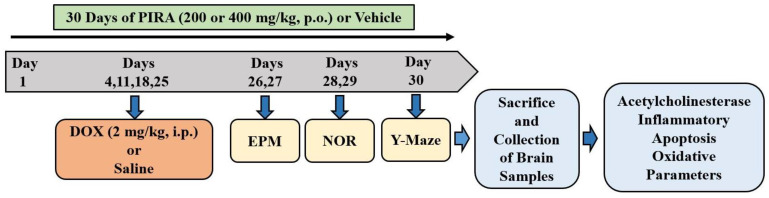
Timeline of the in vivo experiments. Four groups of rats were orally administered with the vehicle or piracetam (PIRA) for 30 days. Except for the control, all groups were injected with four doses of doxorubicin (DOX; 2 mg/kg, i.p.; days 4, 11, 18, and 25) to induce neurotoxicity. For the elevated plus maze (EPM) assessments, the training sessions were conducted on day 26, and retention assessments were analyzed on day 27. The novel object recognition (NOR) test was conducted on day 28 (habituation) and day 29 (training and test sessions), respectively. Both sessions (training and test sessions) of the Y-Maze test were conducted on day 30. At the end of the memory tests, on day 30, all the animals were sacrificed and brain tissues were collected for ELISA tests.

Among four groups, group 1 served as the normal control, and the animals were treated with normal saline (0.1 mL/100 g, p.o.) for 30 days. They also received four doses of intraperitoneal (i.p.) injections of normal saline (0.1 mL/100 g, i.p) on the 4th, 11th, 18th, and 25th days of the drug treatment schedule. Group 2 was considered as the doxorubicin ((DOX), ADRIN^®^, Fresenius Kabi Oncology Ltd., Pune, India) group. The rats were treated with normal saline (0.1 mL/100 g, p.o.) for 30 days and the neurotoxicity was induced with four injections of doxorubicin (2 mg/kg/week, i.p.) once a week for four weeks (4th, 11th, 18th, and 25th days) of the drug treatment schedule [2,18]. Treatment groups (3 and 4) were administered piracetam ((PIRA), Toronto Research Chemicals, Toronto, ON, Canada) in two selected doses (200 mg/kg and 400 mg/kg, respectively, for 30 days) and the neurotoxicity was induced with concomitant administration of doxorubicin (2 mg/kg/week, i.p.) like the DOX group (group 2). PIRA was dissolved in a sterile isotonic saline solution and its doses were selected based on the previous reports [31]. The spatial memory of animals was assessed using an elevated plus maze test (26th and 27th days of drug treatment), novel object recognition test (28th and 29th days of drug treatment), and Y-maze test (30th day of drug treatment). At end of the maze tests, all the animals were sacrificed and the whole brain was collected for various biochemical evaluations (Figure 8).

### 4.3. Assessment of Spatial Memory

#### 4.3.1. Elevated Plus-Maze (EPM) Test

EPM is a neutral behavioral model that is frequently used to assess the memory of rodents. It consists of two enclosed arms and two open arms. During the experiment, the maze is raised 50 cm from the ground, as rodents dislike staying in the open and elevated arm and like to explore and stay in the enclosed arm. Transfer latency (TL), which is the time taken by rats to enter the enclosed arm from any one of the open arms, was estimated on the training day (26th day of drug treatment) as well as on the experiment day (27th day of drug treatment). On the training day, the rats were allowed to explore the maze for 2 min and the retention of memory was evaluated after 24 h [26,27,28].

#### 4.3.2. Novel Object Recognition (NOR) Test

The NOR test has been specifically used to evaluate the cognition ability of rodents in recognition memory for various experimental CNS models. The task was carried out in a wooden box (80 × 60 × 40 cm) that contained two dissimilar objects. Among them, the rectangle box was considered a familiar object, and the cylindrical box was treated as a novel object. As described in earlier research, the total procedure was divided into three different sessions like habituation (28th day of treatment), training, and test (29th day of treatment) phases, and similar protocols were followed throughout the experiment [26,27,28]. During experiment, the exploration times of both familiar objects (FO1 and FO2) were measured in the training session, as were the exploration times of the familiar object (FO1) and the novel object in the test session. Besides, the percentage of the discrimination index was also calculated to explain the capability of animals in the exploration of novelty versus familiarity.

#### 4.3.3. Y-Maze Test

The Y-maze test was used to assess the ability of the rats to recognize the novel arm and their tendency to explore a new place. The wooden Y-maze has three arms with a dimension of 50 (l) × 10 (b) × 30 (h) cm. Each of the arms is at a 120° angle to the other arms. In the training session (30th day of treatment), one arm (novel) was closed and the animals were allowed to explore in the other two arms freely for 5 min. The total number of entries in both know arms were noted in this session. After 4 h, the novel arm was opened and the rats were allowed to explore again for 5 min as a test session. The number of entries by the animals in the known and novel arms were recorded and the percentage of total time spent in the novel arm was also calculated for each animal [26,27,28].

### 4.4. Enzyme-Linked Immunosorbent Assay (ELISA) Using Brain Homogenate

#### 4.4.1. Brain Samples Collection

On day 30, after the last dose of drug treatment and maze tests, all animals were sacrificed by the cervical decapitation method under light ether anesthesia. After sacrifice, the whole brain was immediately removed from the skull and homogenized. The collected homogenate was used to determine the cholinergic and neuroinflammatory biomarkers as described below.

#### 4.4.2. Acetylcholinesterase (AChE)

The AChE estimation was done using the ELISA kit purchased from MyBioSource (Catalog # MBS2709297; MyBioSources Inc., San Diego, CA, USA). The principle of the assay depends on the interaction between the antigen (AChE) and the antibody specific to AChE resulting in color change, which was measured spectrophotometrically (BioTek microplate reader, BioTek Instruments, Santa Clara, CA, USA) at 450 nm.

#### 4.4.3. Inflammatory Markers

The selected inflammatory biomarkers such as prostaglandin E2 (PGE2; Catalog # MBS7606497), cyclooxygenase-2 (COX-2; Catalog # MBS160196), nuclear factor-kappa beta (NF-κβ; Catalog # MBS764450), and tumor necrosis factor-alpha (TNF-α; Catalog # MBS824824) assays were performed using the specific rat ELISA kit purchased from MyBioSources (MyBioSources Inc., San Diego, CA, USA). The basic principle of estimations depends on the reaction between the specific antigen with the biotinylated detection antibody in the presence of horseradish peroxidase-streptavidin (SABC), leading to a color change in the solution. This was measured spectrophotometrically at 450 nm (EL × 800 Absorbance Microplate Reader, BioTek Instruments, Santa Clara, CA, USA) and compared with the standard to determine the concentration.

#### 4.4.4. Apoptotic Proteins

An anti-apoptotic protein B-cell lymphoma-2 (Bcl-2; Catalog # MBS452319) and two pro-apoptotic proteins such as Bcl2 associated X protein (Bax; Catalog # MBS2703209) and cysteine aspartate specific protease-3 (caspase-3; Catalog # MBS729893) were estimated using specific rat ELISA kits from MyBioSources (MyBioSources Inc., San Diego, CA, USA). The assay procedures were followed as per the manufacturer’s protocol and the final absorbance was documented at 450 nm using an ELx800 Absorbance Microplate Reader (BioTek Instruments, Santa Clara, CA, USA).

#### 4.4.5. Oxidative Parameters

Estimation of an oxidative marker, malondialdehyde (MDA; MBS738685), and two antioxidant markers, catalase (CAT; MBS2704433) and glutathione (GSH; MBS775264), were measured by using rat ELISA assay kits obtained from MyBioSources (MyBioSources Inc., San Diego, CA, USA). Analysis was completed by measuring the colour development at 450 nm using an ELx800 Absorbance Microplate Reader (BioTek Instruments, Santa Clara, CA, USA).

### 4.5. Statistical Analysis

The results were stated as mean ± SEM (standard error). The collected data was evaluated using a one-way ANOVA test and the significance level between the groups was analyzed with a Tukey–Kramer post hoc test (Graph Pad version 9.0, GraphPad Software Inc., San Diego, CA, USA). A probability value of 0.05 was considered significant.

## 5. Conclusions

The data from the present study indicated that PIRA treatment of DOX-induced animals decreased the transfer latency in an elevated plus maze test, improved the exploration time as well as the discrimination capability of objects in the novel object recognition test, and increased the number of visits as well as the length of time spent in the arms of the Y-maze test. The observations suggest that PIRA improved the cognitive defects induced by DOX. The nootropic mechanism of PIRA could partly be linked to the reduction of acetylcholinesterase levels, neuro-inflammatory mediator levels, pro-apoptosis proteins levels, and oxidative stress in the brain. Further studies are currently being conducted to explore in more detail the action of PIRA against DOX-induced chemobrain in experimental animals.

## Data Availability

The data presented in this study are available from the corresponding author upon reasonable request.
